# Pellagra—Preliminary Paper

**Published:** 1915-02

**Authors:** Rufus T. Dorsey

**Affiliations:** 802 Empire Building, Atlanta, Ga.


// Journal-Record of Medicine

Successor to Atlanta Medical and Surgical Journal, Established 1855
and Southern Medical Record, Established 1870

**OWNED BY THE ATLANTA MEDICAL JOURNAL COMPANY**
PUBLISHED MONTHLY

*Official Organ Fulton County Medical Society, State Examining Board, Presbyterian Hospital, Atlanta, Birmingham and Atlantic Railroad Surgeons' Association, Chattahoochee Valley Medical and Surgical Association, Etc.*

EDGAR BALLENGER, M. D., Editor
BERNARD WOLFF, M. D., Supervising Editor
A. W. STIRLING, M. D., C. M., D. P. H.; J. S. HURT, B. Ph., M.D.
GEO. M. NILES, M. D., W. J. LOVE, M. D., (Ala.); Associate Editors
R. R. DALY, M. D., Associate Editor
E. W. ALLEN, Business Manager

**COLLABORATORS**

W. F. WESTMORELAND, M. D., General Surgery
F. W. McRAE, M. D., Abdominal Surgery
H. F. HARRIS, M. D., Pathology and Bacteriology
E. B. BLOCK, M. D., Diseases of the Nervous System
MICHAEL HOKE, M. D., Orthopedic Surgery
CYRUS W. STRICKLER, M. D., Legal Medicine and Medical Legislation
E. C. DAVIS, A. B., M. D., Obstetrics
E. G. JONES, A. B., M. D., Gynecology
R. T. DORSEY, Jr., B. S., M. D., Medicine
L. M. GAINES, A. B., M. D., Internal Medicine
GEO. C. MIZELL, M. D., Diseases of the Stomach and Intestines
L. B. CLARKE, M. D., Pediatrics
EDGAR PAULLIN, M. D., Opsonic Medicine
THEODORE TOEPEL, M. D., Mechano Therapy
A. W. STIRLING, M. D., Etc., Diseases of the Eye, Ear, Nose and Throat
BERNARD WOLFF, M. D., Diseases of the Skin
E. G. BALLENGER, M. D., Diseases of the Genito-Urinary Organs

VOL. LXI. ATLANTA, GA., FEBRUARY, 1915. NO. 11

### PELLAGRA—PRELIMINARY PAPER.

### A THEORY AS TO ORIGIN—A SUGGESTION AS TO THERAPY.

BY RUFUS T. DORSEY, M. D.

Pellagra has intensely interested the writer since 1905, and all available literature has been read and studied by him since that time. Many attractive ideas have been advanced during this period, but from out the mass nothing of real import is to be discerned unless it be the dietetic researches of the United States Public Health Service. The literature has been enriched conspicuously, but the incidence of the malady is in no wise curtailed nor has its progress been stayed appreciably.

A great interest and a large clinical experience coupled with the optimistic and tenaciously adhered to belief that pellagra was curable led the author gradually to evolve a theory and to suggest a line of treatment predicated on that conception of its origin.

I believe that pellagra is a nutritional disease—a chronic intoxication resulting from an unbalanced diet too long continued—that such a ration induces a perversion of metabolism which permits of the formation and the accumulation in the economy of a "fatty acid lipoid"—that this body is toxic or rendered so, and has great affinity for nerve structures. This substance is conceived to be the inciting cause of pellagra.

The particular class of foods responsible for the poisonous lipoids is believed to be the carbohydrates and non-leguminous vegetables taken in excess over a period of time, coupled with a deficient intake of the proteid and leguminous types. The offending articles themselves are of good quality; perhaps nontoxic, wholesome before and after preparation for the table. In other words, the trouble is not created by bad food, but by too much of one class of good food and after intake. This lipoid accumulates in the system, at times latent, at other times actively toxic, being precipitated or augmented by a change of body chemistry which may result from various influences and occurrences, as fright, grief, etc.

The idea of a residual remittently precipitated lipoid as the cause of pellagra was not arrived at as a result of pathological or physiological chemical investigation, nor of any attempts as yet to seek any particular lipoid—neither ability or facilities are possessed by me for work in this highly specialized branch of medicine. The idea was prompted solely by clinical observation of pellagra, and here it is appropriate to direct your attention to just a few such indices.

First: Perhaps more than one hundred cases, for various surgical conditions have gone to the operating table in Atlanta during the last nine years without subjective or recognizable objective symptoms of pellagra, and then in from one to eight days afterwards have suddenly and violently presented all the salient symptoms of acute pellagra in its most classic form. Shock is offered to explain such eruption, and yet shock was in no wise and at no time diagnosed or suspected prior to the outbreak in many cases. This basis was not altogether satisfying. A theory only, but more logical, is that a retained lipoid is changed, solved or in some way precipitated by the ether or chloroform used for the anesthesia. All body cells certainly are reached when a sufficiency of these volatile substances are used for complete unconsciousness—a degree of saturation, in fact.

Lipoids are composed of fat-like substances, insoluble in water, hot or cold, acids, diluted or concentrated, alkalies, weak or caustic—in fact, solved by few things only—none of which so far as I know ever used medically in treating pellagra. The failure to use any lipoid solvent in efficacious doses over sufficient time may explain why all prior therapy has been so impotent. Ether and chloroform are ready solvents of lipoids, and the conversion or precipitation by these substances of such lipoids may occasion the toxic phenomena.

Cases of pellagra thus flared up by ether and chloroform pursue various courses. Some are so overwhelmed by the enormous amount of toxic acid liberated that they succumb before sufficient is eliminated or settles back in its original lipoid state. Others where quantity is not so great obtain elimination and pass on to restored health, and apparently a clinical cure. Some have remained well for several years, to my personal knowledge. All grades and courses between these extremes have been observed.

The idea was further augmented as a result of the following observation: The only medicines ever used by me that seemed frequently to produce from little to much good in most cases, is a chologogue tablet composed of Ipecac protoiodide, phenolphthalein and sodium-glycocholate. I originated this in order to get what I considered the proper dose of each component and for use as a laxative, particularly in intestinally toxic cases presenting (as I think) vicid bile, costiveness and icteroid skins. It has much merit in cases of this kind and has also helped my pellagrins.

Now lipoids are solved also by bile acids and sodium glycocholate is the acid salt or bile that serves to thin this excretion. Cholesterine, a typical lipoid, is precipitated from vicid bile, and in the presence of gall bladder infection forms gall stones. Perhaps if this acid were of normal quantity, the bile would be fluid and prevent such an occurrence by retaining the lipoid cholesterine in solution.

The liver is a great detoxifier and this function is supposedly exerted by its normal secretions.

From all this, and not so far fetched, is the deduction that that tablet benefits chiefly by reason of its lipoid solvent contents and that retention is prevented by elimination thus favored or secured.

The third point is that the body odor detected in florid pellagra suggests a fatty-acid—the stools emit a peculiarly uncommon and persistent odor. The odor of fatty acids, as Butyric and acetic—oxybutyric—is often promptly distinguished.

### TREATMENT.

Treatment is divisible into three considerations: The medical, the diet and the symptoms.

Under medical, our efforts are—FIRST—to bring this body into solution and then obtain its elimination. As to the first phase: The remedies at once to suggest—are the agents that possess the property of dissolving of lipoids and are themselves not poisonous to the individual. In appropriate medical doses ether, chloroform or benzol may be administered in several forms and by several different methods—all are quick solvents of lipoids in the test tube. Volatile oils, gasoline, coal oil and a few other fluids bring the lipoids promptly into solution, but for various reasons are unavailable for internal use. Orally we may administer ether (sulphuric or acetic) as such in doses from one-half to two drachms—more acceptably, however, as Hoffman's anodyne or Spiritus ætheris nitrosi in the usual doses. Chloroform as such in 5 to 15 drops—or as chloroform water. Benzol may be given in 5 to 15 drops in oil, emulsion or in capsules, all of course, to be given well diluted and three times a day. The particular formula that I have used in my cases is as follows:

| | |
|---|---|
| Spts. Aetheris Compositus ...... | ½ dram. |
| Chloroformi ................. | 5 drops. |
| Spts. Ammonia aromat. ........ | 20 drops. |
| Tr. Lavendula q. s. ............ | 1 dram. |

This dose does not appeal to the palate, but is not so repugnant as to require persuasion on the attendant's part.

Hypodermically, ether or benzol may be used, but are local irritants and should be diluted with alcohol or some bland oil, such as sterilized liquid aboline—20 drops of the first and 10 drops of the latter may be given daily. Ether as often as three times a day or more as its action is so transient. When using benzol watch the blood.

By rectum the ether-oil-colonic procedure may be utilized. The ether-oil ratio should be reversed, however, and of this mixture only one to two ounces should be used, as anesthesia is undesirable and should be avoided. For fear of local irritation once or twice daily should not be exceeded. When treatment is long continued the method should be varied. Stages of illness will at times render imperative variations as to methods.

To accomplish the second phase all avenues of elimination are to be utilized. Liberal quantities of water to encourage kidney activity. Exercise when permissible—this stimulates and deepens respiration, whips up secretions and improves circulation and makes for a more complete oxidation of food. Hot baths unless contra-indicated yield their quota.

The excretion that most concerns us, however, is the bile. Insufficiency here with costiveness is definitely the pellagrin's enemy and on the other hand, drastic purgatives are both perturbing and harmful. Remedies having chologogue properties are here most beneficial, in moderately effective doses. The remedy that I personally use for this trouble is the tablet previously mentioned, and my reasons are given. Other combinations might be just as good in every respect.

The second and the most important consideration is that of balancing the diet. Were the medicinal agénts suggested proven to be specifics as to their solvent qualities, the end of pellagra would be much the same if we did not interdict the formation of the poison dietetically. Rich and poor pellagrins are (so to speak) nourished out of the same pot. The former from reluctant choice, the latter from sheer necessity. Interpreted, means that the poor are unable financially to purchase the desired proteids, and the rich almost invariably (in my experience) have possessed some kind or degree of abnormality referrable to the buccal-gastro-enteric tract which they seek to overcome by a bland monotonous and deficient diet. The meat proteids, the most important perhaps, are usually first to be eschewed. Indican found in the urine by physicians may have started some pellagra-wards on the grounds of proteid putrification—omission of this type of food naturally followed.

A good working clinical diet rule is to have sufferers now eat as much proteid as they did carbohydrates, and as little carbohydrates now as they did proteids. This rule to prevail only until deficiency is wiped out, as expressed by a dissipation of the intoxication, by marked clinical improvement or a satisfactory restoration of well being generally. Then a normally balanced diet should be instituted and the same should preserve this satisfactory state. Eggs, milk, fresh lean meats of all kinds—brains, liver, etc., cheese, legume, fruits (fresh and dried) are to be urged especially. Soups, broths, juices, extracts made from the above articles are to be used when urgent barriers exist as to the solid diet. Eggs and milk are rarely ever restricted. Excess fats, sweets and non-leguminous vegetable are apparently a cause of pellagra, and are to be practically omitted for the time, and to err on the safe side if we err at all, and moreover out of deference to the opinion of many good observers, corn, its products and preparations are left off as long as there is any manifestation of the disease.

Goldberger's article dealing with foods in pellagra impresses me as more nearly accurate than anything I have ever read. I herewith refer you to same (Public Health Reports, re-print No. 228). A pellagrin may dig his grave with his teeth and with his teeth he may also arise therefrom. Restricted food is their downfall—and a more liberal food and better food is their savior, if they are not too far gone to accept salvation.

The third consideration is the symptomatic treatment. Pages could be consumed upon this topic, but such is unnecessary as they would be trite and hackneyed.

Only on one phase do I desire to comment, and this is the free hydro-chloric acid per cent in stomach contents. Not invariably but surely in the vast majority, pellagrins show a hypo or an anacidity. This acid and its concomitant enzyene have materially to do with the proper digestion of proteids, which are now so important, and sooner or later we ought artificially to supply same and that in appropriate doses. 2-10 per cent free acid is found after an Ewald meal, but 4-10 per cent is found in juice obtained pure and unmixed. Now 4-10 per cent is roughly 2 drops of acid to the ounce and if approximately 5 pints are secreted in 24 hours (estimated). Calculation shows that we have about 160 drops of strong hydro-chloric acid produced daily. This quantity of strong acid is equivalent to 480 drops, or 1 ounce, of dilute acid—the form usually administered. This is a large amount, never given I know, but the normal man uses up about that quantity in digestion every 24 hours. (The exact amount of juice is unproven.)

While I would not advocate attempts to supply the full amount, it is desirable to emphasize that the doses as usually given are not sufficient fully to serve the purpose intended. In curable cases, ordinarily appreciable improvement may be obtained in from 3 to 6 weeks—decided benefit in 6 to 10 weeks, provided the medicinal dietetic and symptomatic remedies are wisely utilized. Clearly the emphasis is to be placed on obtain ing the properly balanced and varied diet. It is inconceivable that any permanent results could obtain without it.

It takes more than several swallows to make a summer, and I am keenly aware that one as intensely interested in pellagra as I am is very prone to allow his enthusiasm to warp his judgment. Erroneous deductions and falacious conclusions just creep in unawares, in spite of this knowledge, sometimes.. Hand-running many cases in the past have seemingly been helped by certain medicines, and yet we now know they had no virtue whatsoever in actuality. A detailed comment on the very few cases I have handled along this line would be useless, and I defer such on that ground. However, three cases that previously resisted betterment have coincidentally with this therapy made gratifying progress—not so rapid, I grant, but they are now on what appears to be secure ground. Four additional cases have been added quite recently, and will be watched with much conservatism. In conclusion, let me state that this paper is not on the cause and cure of pellagra. The caption distinctly reads, "Pellagra, a Theory as to Origin, and a Suggestion as to Therapy."

By the end of May I expect to be far enough along in my investigations to present you with a clinical report of cases or to cast the whole among the discards. Drs. Eberhart and Funke have kindly agreed to contribute their valued services in physiological and pathological chemistry.

802 Empire Building, Atlanta, Ga.

